# The Versatile Pseudomonas aeruginosa Biofilm Matrix Protein CdrA Promotes Aggregation through Different Extracellular Exopolysaccharide Interactions

**DOI:** 10.1128/JB.00216-20

**Published:** 2020-09-08

**Authors:** Courtney Reichhardt, Holly M. Jacobs, Michael Matwichuk, Cynthis Wong, Daniel J. Wozniak, Matthew R. Parsek

**Affiliations:** a Department of Microbiology, University of Washington, Seattle, Washington, USA; b Department of Microbial Infection and Immunity, The Ohio State University, Columbus, Ohio, USA; c Department of Microbiology, The Ohio State University, Columbus, Ohio, USA; Geisel School of Medicine at Dartmouth

**Keywords:** *Pseudomonas*, adhesin, bacterial communities, biofilm

## Abstract

Pseudomonas aeruginosa is an important pathogen that causes chronic infections that involve multicellular aggregates called biofilms. Within biofilms, bacteria are surrounded in a protective extracellular matrix of proteins, exopolysaccharides (EPS), and DNA. A key P. aeruginosa matrix protein is an extracellular adhesin called CdrA, which promotes aggregation by binding to the EPS Psl and via CdrA-CdrA interactions. We hypothesized that because of its ability to bind Psl, CdrA would be important only for strains that use Psl as the primary EPS (e.g., the laboratory strain PAO1). Thus, we predicted that *cdrA* might be dispensable for biofilm formation by strains that do not utilize Psl (e.g., the laboratory strain PA14). Instead, we observed that *cdrA* deletion strains exhibited biofilm defects, regardless of their EPS dependencies. We screened a panel of clinical and environmental P. aeruginosa isolates for the presence of the *cdrA* allele and production of CdrA protein. All isolates that we tested contained the *cdrA* allele, and these alleles had minimal sequence variation compared to the reference PAO1 *cdrA* gene. Additionally, all isolates except one produced detectable CdrA protein. We investigated the possible mechanisms of CdrA-promoted biofilm formation in these strains where Psl is not dominant, and we discovered that CdrA binds to Pel. Although Psl and Pel chemical structures are distinct, this appears to be a specific interaction, since previous work has shown that CdrA binds discriminately to other EPS. Our findings provide new understanding of biofilm formation across P. aeruginosa isolates and emphasize the versatility of CdrA.

**IMPORTANCE** Depending upon the strain, Pseudomonas aeruginosa can use different exopolysaccharides (e.g., Psl, Pel, and alginate) to build its biofilm matrix. Previously, we demonstrated that the biofilm matrix protein CdrA binds to Psl, promoting biofilm formation and aggregate stability. As such, it was thought that CdrA might be important for biofilm assembly only in strains that rely upon Psl. However, past studies indicated that CdrA can interact with monosaccharides not present in Psl, including *N*-acetylglucosamine, a constituent of another EPS called Pel. We discovered that CdrA also binds to Pel and promotes biofilm formation by strains in which Psl is not dominant. Thus, our findings suggest that CdrA plays a common role as a biofilm matrix cross-linker across P. aeruginosa isolates with different EPS.

## Abstract (precis)

Depending upon the strain, Pseudomonas aeruginosa can use different exopolysaccharides (e.g., Psl, Pel, and alginate) to build its biofilm matrix. Previously, we demonstrated that the biofilm matrix protein CdrA binds to Psl, promoting biofilm formation and aggregate stability. As such, it was thought that CdrA might be important for biofilm assembly only in strains that rely upon Psl. However, past studies indicated that CdrA can interact with monosaccharides not present in Psl, including *N*-acetylglucosamine, a constituent of another EPS called Pel. We discovered that CdrA also binds to Pel and promotes biofilm formation by strains in which Psl is not dominant. Thus, our findings suggest that CdrA plays a common role as a biofilm matrix cross-linker across P. aeruginosa isolates with different EPS.

## INTRODUCTION

Bacteria form multicellular communities called biofilms (1). Within biofilms, bacteria are enmeshed in a self-produced extracellular matrix. The composition of biofilm matrix varies depending upon bacterial species or even strain but typically includes some combination of exopolysaccharides (EPS), proteins, and extracellular DNA (eDNA). In biofilms, bacteria are protected from harsh environmental conditions and medical treatments, including antibiotics (2–4). Pseudomonas aeruginosa is a paradigm organism for the study of biofilms and causes chronic, biofilm-related infections, including wound infections, otitis (ear infections), urinary tract infections, ventilator-associated pneumonia, and cystic fibrosis (CF) lung infections (5–7).

P. aeruginosa can produce at least three types of EPS, which have all been implicated in the biofilm mode of growth. Mucoid strains are commonly isolated from CF infections and are characterized by the overproduction of alginate, which serves as the primary matrix scaffold (8). Nonmucoid strains usually rely on the EPS Psl and/or Pel as their matrix scaffold(s) (9). Psl is a neutral, mannose-rich EPS (10), and Pel is a cationic polysaccharide composed of *N*-acetylglucosamine (GlcNAc) and *N*-acetylgalactosamine (GalNAc) (11). A study by Colvin et al. investigated the role of EPS in biofilm formation from a panel of nonmucoid environmental and clinical isolates of P. aeruginosa (9). The isolates were found to vary in their dependence on either Psl or Pel for producing biofilm aggregates. From the findings of this study, nonmucoid P. aeruginosa strains can be categorized based on their EPS biofilm matrix dependence as (i) those in which Pel is dominant, (ii) those in which Psl is dominant, (iii) those in which the EPS is redundant (i.e., both Pel and Psl are produced and are individually sufficient to produce biofilm), or (iv) matrix overproducers. The panel of strains used in this study and their classification are presented in Table 1.

**TABLE 1:** P. aeruginosa isolates with different EPS types used in this study

EPS class (type) and strain	Description^*a*^	Reference
I (strain with matrix where Pel is dominant): PA14	Laboratory strain	18
		

II (strains with matrix where Psl is dominant)		
PAO1	Laboratory strain	17
S54485	UTI isolate	16
X13273	Blood isolate	16
E2	Tomato plant isolate	16
62	Soil isolate	16
X24509	UTI isolate	16

III (strains with matrix with redundant EPS)		
MSH3	Water isolate	16
MSH10	Water isolate	16
T56593	Ear infection isolate	9

IV (matrix overproducers)		
CF127	Cystic fibrosis isolate	16
19660	Cornea/ocular isolate	16

a UTI, urinary tract infection.

**FIG 1:**
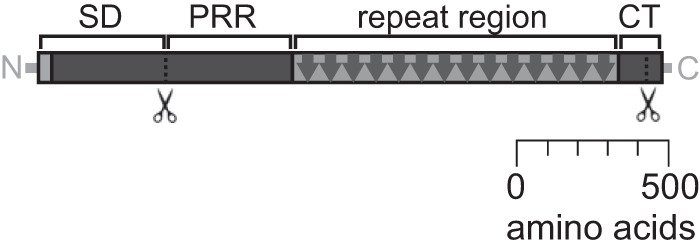
Schematic of CdrA. CdrA is a large (2,154-amino-acid), repetitive protein. Homology modeling predicts that CdrA has several structural motifs, including a secretion domain (SD), prerepeat region (PRR), repeat region, and C-terminal region (CT) (12). The repeat region of CdrA contains 14 repeats of 81 amino acids per repeat. As indicated in the schematic, CdrA can be cleaved near both its N and C termini. CdrA is released from the cell surface if cleaved at a C-terminal TAAG site by the periplasmic protease LapG (13, 14).

In addition to EPS, the P. aeruginosa biofilm matrix contains proteins that have structural, protective, or other functional roles (2, 12). CdrA was the first P. aeruginosa biofilm matrix protein to be reported, and it plays a structural role in biofilm aggregates (12). CdrA is a large protein adhesin that is the cargo of a two-partner secretion system encoded by the *cdrAB* operon. As indicated in the schematic in Fig. 1, CdrA has several predicted domains, including an N-terminal signal sequence, an N-terminal prerepeat region, and a repeat region that is predicted to be beta-sheet rich (12). CdrA is found in both cell-associated and released forms. Under conditions of low cyclic di-GMP, the periplasmic protease LapG cleaves cell-associated CdrA at a C-terminal TAAG site to release CdrA from the cell surface (13, 14). CdrA promotes biofilm stability and aggregation using CdrA-Psl (12) and CdrA-CdrA (15) interactions. These interactions protect against proteolytic degradation in the extracellular environment and mechanical disruption, respectively (15). CdrA-dependent aggregation can be impaired through the exogenous addition of several sugars, including mannose (a constituent monosaccharide of Psl), *N*-acetylglucosamine (a constituent monosaccharide of Pel), l-fucose, and d-fructose, and this blocking is presumed to occur via competitive binding (12). This suggests that CdrA binds to a range of biomolecules in addition to Psl.

Previously, due to its ability to bind Psl, CdrA was thought to be important only for strains that produced a Psl-rich biofilm matrix (e.g., PAO1) (12). However, based upon our recent findings that EPS-independent CdrA-CdrA interactions promote aggregation (15) and the range of CdrA sugar-binding partners identified in the aggregation assay (12), we hypothesized that CdrA may play a role in P. aeruginosa isolates with different EPS reliance (such as strains in which Pel is dominant or the EPS are redundant). We tested this hypothesis by first verifying that isolates, regardless of EPS reliance, are able to produce CdrA. We then evaluated if CdrA was required for biofilm formation and found that in general, *cdrA* mutant strains produced structurally defective biofilms relative to their wild-type counterparts, including PA14, which is strictly Pel dependent. This observation led to the finding that the EPS Pel is a novel binding partner for CdrA. Together, these results highlight the versatility of CdrA and its central importance as a structural component of the P. aeruginosa biofilm matrix.

## RESULTS AND DISCUSSION

### P. aeruginosa isolates produce CdrA of different sizes.

**FIG 2:**
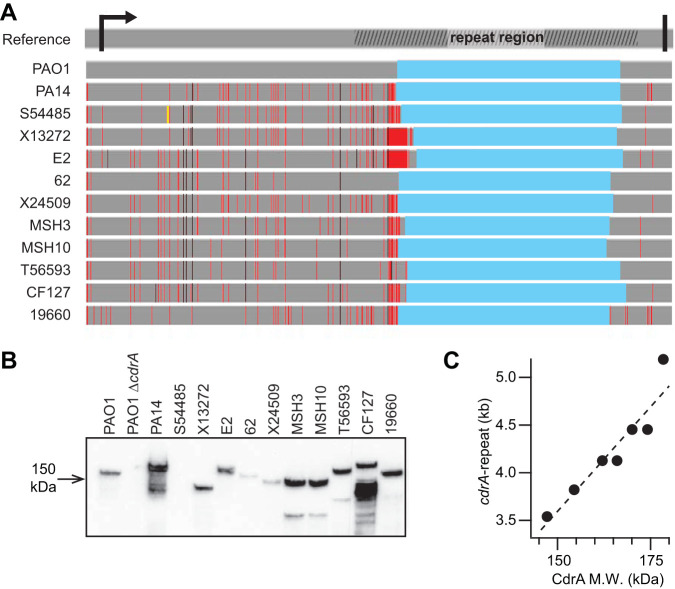
P. aeruginosa isolates produce CdrA of various sizes. (A) P. aeruginosa isolates have nucleotide changes in *cdrA* compared to PAO1. Nucleotide sequences of *cdrA* from 12 strains were aligned to the reference PAO1 *cdrA* gene sequence (top line, obtained from pseudomonas.com). The arrow and thick black line indicate the start and stop codons of *cdrA*, respectively. The hashed area on the reference line indicates the repeat region. In aligned sequences, gray indicates conserved residues, red lines indicate base pair changes, black lines indicate base pair changes that result in predicted amino acid changes from the reference protein, the yellow line indicates a nonsense mutation, and blue indicates a region that was unable to be sequenced. Nucleotide alignment was completed using MAFFT v7. (B) Anti-CdrA Western blot analysis of clinical isolates showed that most isolates make CdrA, with the exception of S54485, and the size of CdrA varied. (C) The *cdrA* repeat size linearly correlated with CdrA protein size, as indicated by the dashed fit line (*r*^2^ = 0.8882). Some of the isolates had identical CdrA sizes and corresponding *cdrA* repeat sizes, and some points on the plot are overlaid.

We surveyed several P. aeruginosa environmental and clinical isolates (9, 16–18) for the presence of a chromosomal copy of *cdrA* and whether it is expressed under standard lab culturing conditions. The regions outside the *cdrA* repeat region were amplified and sequenced, and very few base pair differences were noted using the PAO1 *cdrA* sequence as a reference (Fig. 2A; see Fig. S1 in the supplemental material). These regions include the N-terminal region with the predicted signal for Sec-mediated protein secretion (12) and the C-terminal region, which is involved in tethering and proteolytic release of CdrA from the bacterial cell (13, 14). Amplification and sequencing of the entire *cdrA* gene were challenging due to its size and the stretch of repetitive sequences that encode the repeat region of CdrA (19). To gain some information about the repeat region, we designed primers to amplify the entire *cdrA* repeat region and quantified its size. We found that its size varied from 3.54 to 5.19 kb. For reference, the amplified region of *cdrA* from the reference PAO1 strain is 4.05 kb (Fig. S2). Differences in CdrA size also were observed by Western blot analysis (Fig. 2B). All isolates produced CdrA under the growth conditions tested with the exception of S54485, which has a nonsense mutation that is expected to prematurely truncate CdrA at amino acid 235. For several isolates (e.g., PA14, MSH3, MSH10, T56593, and CF127), multiple bands were observed by Western blotting. Based on previously reported findings, we predict that these are due to proteolysis of CdrA by LasB (15). The different *cdrA* repeat sizes correlate well with CdrA protein size as estimated by SDS-PAGE analysis (Fig. 2C). It is unclear whether these size differences translate to a functional consequence for the protein. However, one might speculate that varying CdrA size may impact its binding affinity and/or the density of the cross-linked extracellular matrix mesh.

### CdrA promotes biofilm aggregation across isolates.

**FIG 3:**
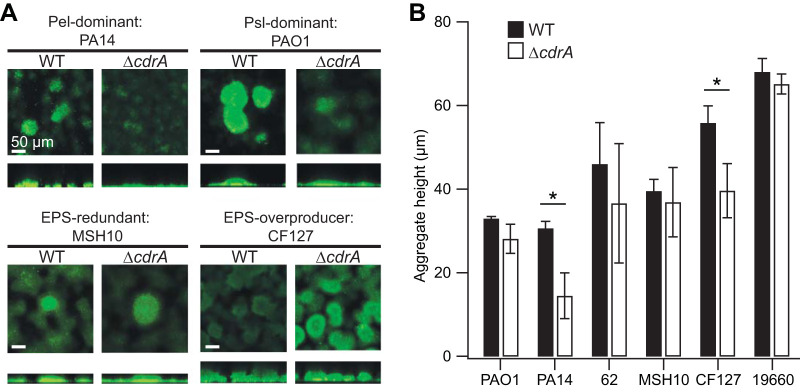
CdrA promotes biofilm aggregation in flow cells. (A) Representative flow cell biofilms of several isolates stained with Syto9 and imaged using confocal laser scanning microscopy (CLSM). In general, the Δ*cdrA* mutants formed shorter biofilm aggregates than their wild-type (WT) counterparts. (B) The difference in aggregate height was quantified. Error bars represent the standard deviations for 3 or 4 biological replicates. The values for each biological replicate were obtained from measurements of 4 to 8 aggregates per flow cell (*, *P* < 0.05 [*t* test]).

CdrA is critical for robust biofilm formation by PAO1, which has been attributed to CdrA-Psl interactions (12). Given that the size of CdrA varied across the isolates, it was possible that CdrA would not be functional in all of the strains. For example, lower-molecular-weight CdrA might not have enough surface area to promote homophilic CdrA-CdrA interactions or might not have retained EPS binding sites, both of which would influence the ability of CdrA to promote aggregation. To test whether CdrA is functional across isolates that rely upon different EPS to form biofilms, we tested the impact of the Δ*cdrA* mutation in the two commonly used laboratory strains, PAO1 (a strain where Psl is dominant in the matrix) and PA14 (Pel is dominant), as well as the clinical and environmental isolates 62 (Psl is dominant), MSH10 (matrix EPS are redundant), CF127 (matrix overproducer), and 19660 (matrix overproducer). All of these isolates were shown by PCR and Western blot analyses to have the *cdrA* gene and produce CdrA (Fig. 2; Fig. S3). Initially, we tested the impact of a Δ*cdrA* mutation on static biofilm formation, and as has been previously reported for PAO1 (12), we observed that the deletion did not significantly impact adherent biomass (Fig. S4). This result could be due to EPS masking the impact of CdrA, as has been previously reported for biofilm protein adhesins (20), or because CdrA does not strongly influence biofilm formation in a static system not subjected to shear. Therefore, we tested the impact of a Δ*cdrA* mutation on biofilm aggregate structure when the cells were grown under fluid shear (Fig. 3A). The wild-type (WT) aggregates differed from one another both structurally and in the amount of adherent biomass. With the exception of MSH10, we observed a trend in which the Δ*cdrA* mutants appeared to be reduced in biofilm aggregation, but the extent of this defect varied.

To quantify the microscopy findings, the average biofilm aggregate height was measured for each strain and its corresponding Δ*cdrA* mutant (Fig. 3B), and in general, the Δ*cdrA* mutants formed shorter aggregates that did not extend into the lumen of the flow channel, where shear effects are most pronounced (*P* < 0.05 for PA14 and CF127; differences for all other strains were not statistically significant). Thus, we propose that CdrA promotes the formation of stable aggregates able to resist mechanical disruption as a consequence of fluid shear. We verified that the observed diminished biofilm aggregate formation was not due to a growth defect of the Δ*cdrA* mutant strains (Fig. S5). For the Pel-dependent strain PA14, the impact of a Δ*cdrA* mutation was dramatic, with very little biomass that adhered to the flow cell coverslip. The mutant phenotype could be complemented by supplying *cdrA* in *trans* (Fig. S6). Interestingly, the Δ*cdrA* mutation was most deleterious to biofilm formation in strains PA14 and CF127, which produce the largest CdrA (Fig. 2). This suggests that CdrA with a longer repetitive region (Fig. 1) may be better able to promote aggregation, and our laboratory is currently exploring this possibility further. Also, these strains, PA14 and CF127, make more CdrA (based on static biofilm conditions [Fig. 2B]) and thus may be more reliant on the matrix protein. Finally, as previously reported, the reliance of both PA14 and CF127 on Pel to form flow cell biofilms was greatest out of the isolate panel, which implies a strong correlation between CdrA dependence and Pel dependence on biofilm formation (9). Overall, these results support the idea that CdrA is important for biofilm aggregate production in a range of strains that vary in biofilm EPS usage.

### CdrA binds to the EPS Pel.

**FIG 4:**
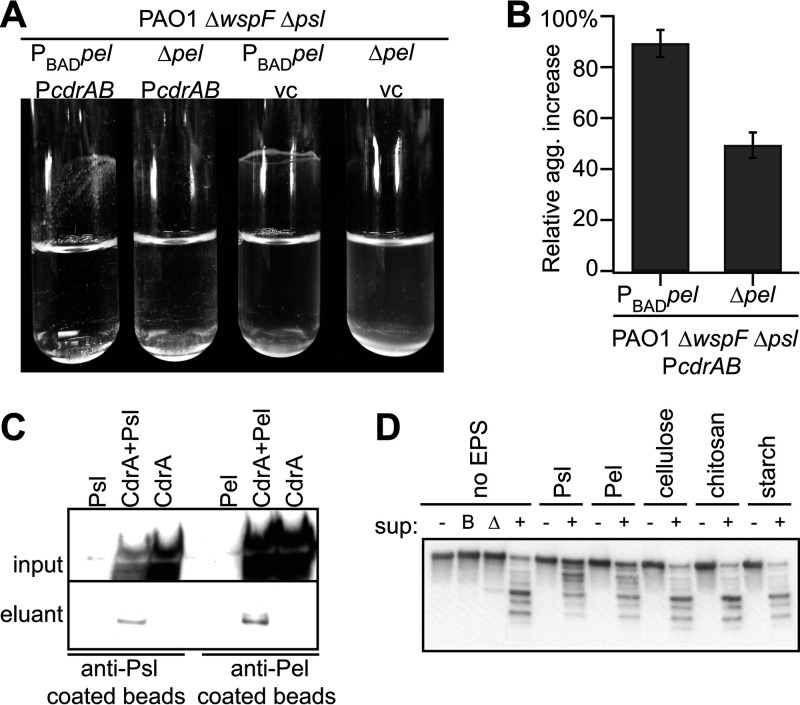
The EPS Pel binds to CdrA. (A and B) Simultaneous overexpression of *cdrAB* and *pel* results in aggregation in liquid culture. (B) Data are means from three replicates, and error bars indicate standard deviations. (C) CdrA was detected in the eluant following a coimmunoprecipitation when both Psl and CdrA were present in culture supernatants and when both Pel and CdrA were present in culture supernatants. (D) Anti-CdrA Western blot analysis shows that both Psl and Pel, but not cellulose, chitosan, or starch, protect CdrA from degradation by P. aeruginosa supernatant (sup) proteases. Intact secreted CdrA, that is, CdrA not treated (−) with supernatant, is detected at approximately 150 kDa. The negative controls, boiled supernatant (lane B) and Δ*lasB* supernatant (lane Δ), do not proteolyze CdrA.

It was previously determined that CdrA binds to Psl (12). Based upon the microscopic analyses of the *cdrA* mutants, particularly in strain PA14, which lacks the genetic capacity to produce Psl (Fig. 3), we hypothesized that CdrA may also bind to Pel. As an initial test of this, we transformed the Pel overexpression strain PAO1 Δ*wspF psl* P_BAD_*pel* with P_BAD_*cdrAB*. We observed that expression of CdrA increased aggregation, and the effect of CdrA was amplified when it was expressed with Pel. This finding suggests that the matrix components CdrA and Pel interact (Fig. 4A and B; Table S1; Fig. S7). Supporting this hypothesis, a coimmunoprecipitation assay determined that CdrA binds to Pel (Fig. 4C). As additional evidence of CdrA-Pel interactions, we demonstrated that CdrA-Pel interactions protect CdrA from proteolysis by the P. aeruginosa protease LasB (Fig. 4D). This was similar to our previous study demonstrating that CdrA-Psl interactions could also protect CdrA from LasB cleavage (15). The capacity of CdrA to bind Pel was likely overlooked in the past due to our lack of knowledge regarding the structure of Pel (it contains the CdrA-binding monosaccharide *N*-acetylglucosamine) and the unavailability of Pel-specific antisera (Fig. S8) (12). Since we do not yet have biochemical data investigating the binding of purified CdrA and Pel in the absence of other biomolecules (e.g., proteins, lipids, etc., present in the culture supernatant), it is possible that some other extracellular biomolecule facilitates the observed interactions between CdrA and Pel. Alternatively, CdrA may facilitate the binding of another molecule that directly binds Pel. The question of whether Psl and Pel bind to different CdrA motifs remains unanswered and is an area of ongoing study in our laboratory.

Since Pel is similar to the EPS produced by other pathogens, specifically, the cationic EPS galactosaminogalactan (GAG) produced by Aspergillus fumigatus (21, 22), our findings raise the possibility that CdrA binds to heterologous EPS types produced by other pathogens. This may promote the assembly of multispecies biofilm aggregates, with important clinical consequences, as both P. aeruginosa and A. fumigatus are lung pathogens. This would be similar to the case in which Candida albicans secretes cell wall polysaccharides that bind to Staphylococcus aureus biofilms (23).

In summary, we found that the P. aeruginosa matrix protein CdrA plays a broader role in P. aeruginosa biofilm formation than was previously appreciated. Specifically, we found that CdrA promotes aggregation across isolates (Fig. 3) and can bind to the EPS Pel in addition to CdrA and Psl (Fig. 4). Prior to this study, the role of CdrA in isolates in which Psl is not dominant was assumed to be negligible (12). As we showed, most surveyed isolates can produce CdrA despite different EPS reliance (such as Pel dominance or EPS redundancy) (Fig. 2), and CdrA is important for aggregation even in isolates in which Psl is not dominant and instead rely either entirely on Pel (i.e., PA14) or on both Psl and Pel (i.e., CF127) to build their biofilms (Fig. 3). None of the isolates tested here were mucoid, which remains an open line of inquiry. Additionally, there appears to be functional flexibility in the size of the CdrA repeat region, which is similar to what has been observed in some other bacterial adhesins (24, 25). Finally, the lack of traditional sugar-binding motifs or lectin domains raises the question of how CdrA engages in these interactions.

## MATERIALS AND METHODS

### Bacterial strains and growth conditions.

Bacterial strains and plasmids used in this study are listed in Table 1 and in Table S2 in the supplemental material. The *cdrA* deletion allele was created using a previously described method (12).

### Sequencing *cdrA*.

Genomic DNA was prepared using the DNeasy blood and tissue kit (Qiagen). *cdrA* repeat regions were amplified using primers CRR_49 and CRR_46 (annealing temperature = 70°C, extension time = 2.5 min). Whole *cdrA* was amplified using primers CRR_45 and CRR_47 (annealing temperature = 59°C, extension time = 3.75 min). All PCRs were performed using Q5 polymerase with GC enhancer (NEB). PCR amplicons for sequencing were excised and purified from agarose gels using the Qiagen gel extraction kit with suggested modifications for purifying long fragments (Qiagen). Sanger sequencing (GENEWIZ) yielded sequence reads which were aligned to the reference PAO1 *cdrA* sequence (www.pseudomonas.com) using MAFFT v7 and www.benchling.com. CdrA protein sequences were made by translating the nucleotide sequence and were aligned to the reference PAO1 CdrA amino acid sequence (www.pseudomonas.com) using Clustal Omega. Primers used for amplification and sequencing are listed in Table S3 in the supplemental material.

### CdrA sample preparation from static biofilms.

Six-well plates were inoculated with 5 ml per well of a 1:20 dilution of a mid-log-phase culture in tryptic soy broth (TSB). The plates were incubated statically at 30°C for 24 h. The entire culture, including the adherent biomass, was suspended using an 18-gauge syringe and passed through the syringe five times. The resulting samples were normalized to an optical density at 600 nm (OD_600_) of 1.0. Cells were pelleted by centrifuging at 16,000 × *g* for 5 min at room temperature, and the pellet was discarded. The supernatants were then analyzed by Western blot analysis as previously described (15).

### Flow cells.

Biofilms were cultivated in flow cell chambers essentially as described by Colvin et al. (9) with some modifications. Flow cells were inoculated from a mid-log-phase TSB culture that was diluted with 1% TSB to an OD_600_ of 0.01 for all strains except for PA14, which was diluted to an OD_600_ of 0.05. Cells were allowed to attach under static conditions to an inverted flow cell for 1 h before induction of flow. Biofilms were grown on 1% TSB for 72 h at room temperature under a constant flow rate (10 ml/h). Biofilms were stained for 15 min with Syto9 green fluorescent nucleic acid stain (5 μM; Life Technologies) for biomass. After staining, flow cells were washed with medium at 10 ml/h for 5 min and then visualized on a Zeiss LSM 800 confocal laser scanning microscope. Images were analyzed with Velocity software (Improvision) and ImageJ.

### Aggregation assays.

Stationary-phase cultures were diluted 30-fold into Jensen’s medium supplemented with 1% (wt/vol) l-arabinose and 300 μM carbenicillin. Cultures were grown at 37°C, with shaking at 225 rpm, for 2 h 15 min. Aggregation was evaluated by visual assessment and the measurement of absorbance at 600 nm. Percent relative aggregation increase was calculated for each of three experiments (one culture tube per strain per experiment) by taking the difference in absorbance of the pBAD*cdrAB* strain and its corresponding vector control strain and dividing by the absorbance of the vector control strain, before multiplying by 100. The average of these three “percent relative aggregation increase” values is displayed on the plot (Fig. 4B), and the errors were calculated through the standard deviation of the replicate samples.

### Coimmunoprecipitation assay.

Stationary-phase cultures were diluted 1:30 and grown for 6 h at 37°C. Cell-free supernatants were obtained by pelleting for 2 min at 16,000 × *g* to remove cells, and then Roche and Halt protease inhibitors were added to the supernatant preparations. Anti-Psl antibodies (MedImmune) (26) and anti-Pel antibodies (11) were cross-linked to magnetic protein G Dynabeads (Life Technologies) according to the manufacturer’s recommendations. Beads were washed twice after antibody binding with phosphate-buffered saline (PBS) containing 0.02% Tween 20. Antibody-coated beads (50 μl) were then incubated for 10 min with 1.2 ml cell-free supernatant preparations and then washed three times with PBS containing 0.02% Tween 20. Proteins coprecipitating with Psl or Pel were eluted with XT sample buffer with reducing agent (Criterion-Bio-Rad) and analyzed by immunoblotting.

### EPS purification.

Stationary-phase cultures were diluted 1:500 in Jensen’s medium supplemented with 2% (wt/vol) l-arabinose and grown overnight, with shaking at 225 rpm, at 37°C. Cells were pelleted by centrifuging twice at 8,300 × *g* for 15 min at room temperature, and the pellet was discarded. Ice-cold ethanol was added to supernatant at a ratio of 3:1 and incubated at 4°C for 1 h. EPS was pelleted by spinning at 8,300 × *g* for 15 min at 4°C, and supernatant was discarded. The pellet was washed three times with ice-cold 95% ethanol, then washed with 100% ice-cold ethanol, and air dried overnight. The sample was tested by immunoblotting for the presence of EPS (11).

### CdrA purification.

CdrA was purified as previously described (15). Briefly, stationary-phase cultures were grown in LB medium supplemented with 1% (wt/vol) l-arabinose and 300 μm carbenicillin. Cells were pelleted by centrifuging twice for 10 min at 5,000 × *g* and discarded. One tablet of Roche protease inhibitor and 100 μl Halt protease inhibitor were added per 25 ml aliquot of supernatant. Supernatant was then concentrated using 100-kDa Amicon filter units. Concentrated supernatant was run on an equilibrated size exclusion column (Sephacryl-300 column), and fractions were collected. Fractions were tested by SDS-PAGE and anti-CdrA Western blot analysis for CdrA.

### Protease susceptibility assay.

The protease susceptibility assay was performed as previously described (15). Briefly, purified CdrA and isolated EPS were incubated together overnight with rotation at room temperature (10 μg CdrA to 30 μg EPS). Sterile water was added to a final volume of 50 μl. As a control, CdrA was incubated with only sterile water. Cell-free supernatants from stationary-phase cultures of PAO1 Δ*wspF* Δ*cdrA* Δ*pslBCD* Δ*pelA* Δ*algD* were added to the CdrA-polysaccharide mixtures. Two parts cell-free supernatant (or boiled supernatant or sterile water) were added to one part CdrA-polysaccharide mixture. Reaction mixtures were incubated at 37°C for 16 h. Commercially available cellulose (Sigma), chitosan (Sigma), and cornstarch (Albertsons) were used.

### Crystal violet assay for static biofilm quantification.

Static biofilm formation was assessed using the crystal violet assay as previously described (9). Static biofilms were cultured in Nunc Bacti 96-well microtiter plates using TSB medium. Cultures were incubated statically for 24 h at 30°C before nonadherent biomass was removed and the crystal violet assay performed.

## Supplementary Material

Supplemental file 1
