# Supplementary material for: The Versatile Pseudomonas aeruginosa Biofilm Matrix Protein CdrA Promotes Aggregation through Different Extracellular Exopolysaccharide Interactions
Source: J Bacteriol. 2020 Sep 8;202(19):e00216-20. doi: 10.1128/JB.00216-20 (PMC7484184; doi:10.1128/JB.00216-20)
Supplement: Supplemental file 1 [file JB.00216-20-s0001.pdf]

SUPPLEMENTAL MATERIAL

Table S1. Optical density values of aggregation assay

	Replicate 1		Replicate 2		Replicate 3	
	$OD_{600\ nm}$	Relative agg. increase	$OD_{600\ nm}$	Relative agg. increase	$OD_{600\ nm}$	Relative agg. increase
P_{BAD}***pel*** P_{BAD}***cdrAB***	0.084	81.82%	0.04	92.29%	0.036	93.02%
**Δ*pel*** P_{BAD}***cdrAB***	0.284	54.49%	0.345	47.89%	0.33	45.72%
P_{BAD}***pel* vc**	0.462		0.519		0.516	
**Δ*pel* vc**	0.624		0.662		0.608	

Table S2. Additional strains and plasmids used in this study

***P. aeruginosa* strains**	**Source**
PAO1 Δ*wspF* Δ*psl* Δ*pel*	Jennings *et al*., 2015
PAO1 Δ*wspF* Δ*psl* pBAD*pel*	Jennings *et al*., 2015
PAO1 Δ*wspF* Δ*EPS* Δ*cdrA*	Reichhardt *et al.,* 2018
PAO1 Δ*wspF* Δ*EPS* Δ*cdrA* Δ*lasB*	Reichhardt *et al.,* 2018; this study

**Plasmids**	**Source**
pMJT-1	Borlee *et al*., 2010
pBAD*cdrAB*	Borlee *et al*., 2010
DH5α p-Δ*lasB*	This study
DH5α p-Δ*cdrA*	Borlee *et al*., 2010

Table S3. Primers used in this study

**Primer**	**Sequence (5'-3')**	**Source**
CRR_11	CGAACATCAGCGACGAACAC	This study
CRR_12	AACGGCAGCCTGGCAA	This study
CRR_27	TCTCGTTGGGGTTGAACTGG	This study
CRR_32	ATCGGCGGCGACTTCCA	This study
CRR_45	GGAGGCATGGTCGAGGAAAA	This study
CRR_46	GTACAGTCCCTGGCAACTCC	This study
CRR_47	TGCCTTCGATGCGTATCTCG	This study
CRR_48	TGACCTGGTAGGTGAGGGTC	This study
CRR_49	CGCCTACGTCAACAGTCAGT	This study
HJ_23	CCAGTTCAACCCCAACGAGA	This study

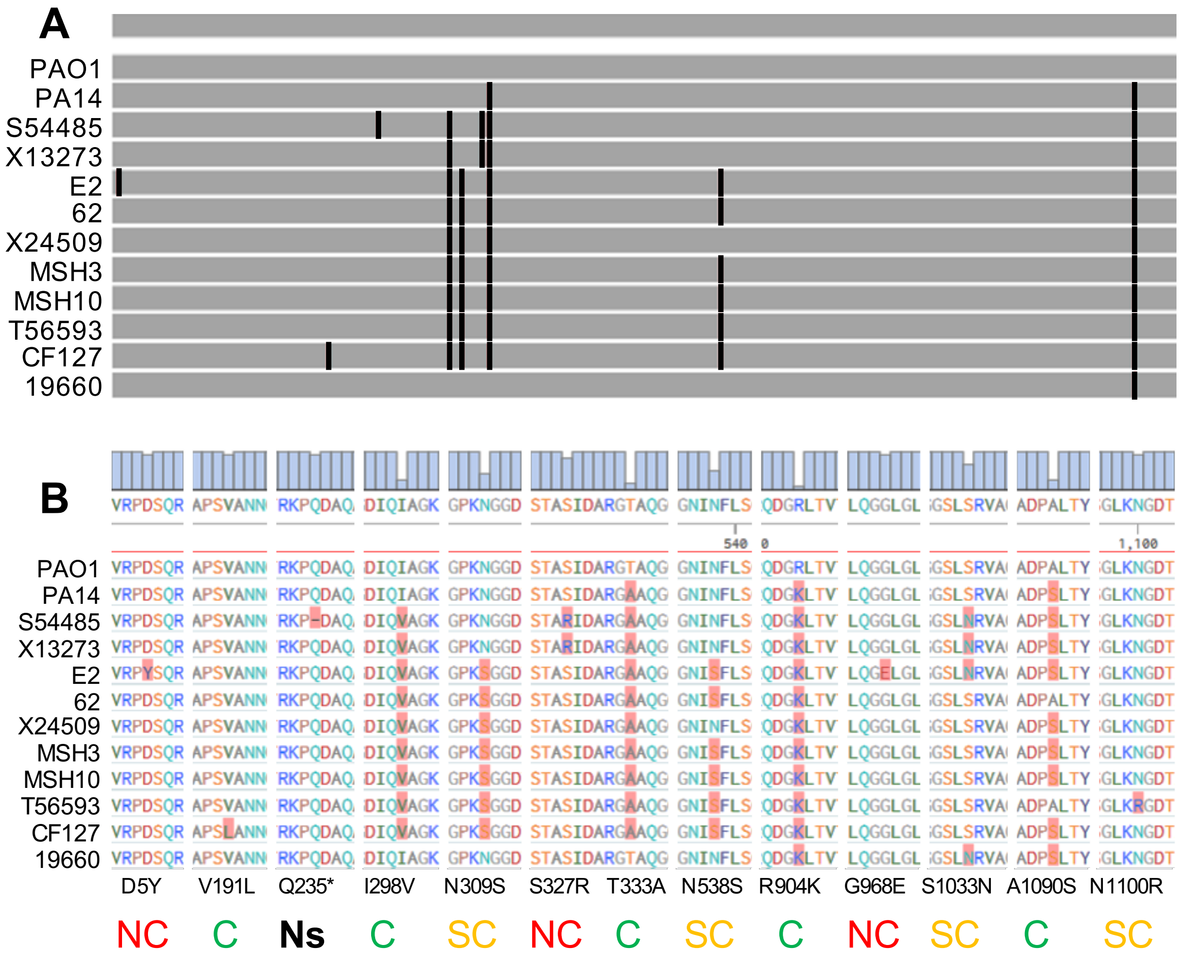

Supplemental Fig 1. *P. aeruginosa* isolates have non-conservative amino acid changes in CdrA compared to PAO1. (A) Protein translation of CdrA from nucleotide sequencing of *cdrA* in 12 strains compared to the reference PAO1 CdrA protein sequence as published on pseudomonas.org (top grey line). Only the N-terminal portion of CdrA is shown. Grey indicates conserved residues; vertical red lines indicate an amino acid change. Protein alignment was conducted using Clustal Omega. **(B)** Individual amino acids (highlighted in red) in clinical isolates differ from the reference PAO1 protein sequence. Conservative (C, green), semi-conservative (SC, yellow), non-conservative (NC, red), and nonsense (Ns, bold) amino acid changes are noted.

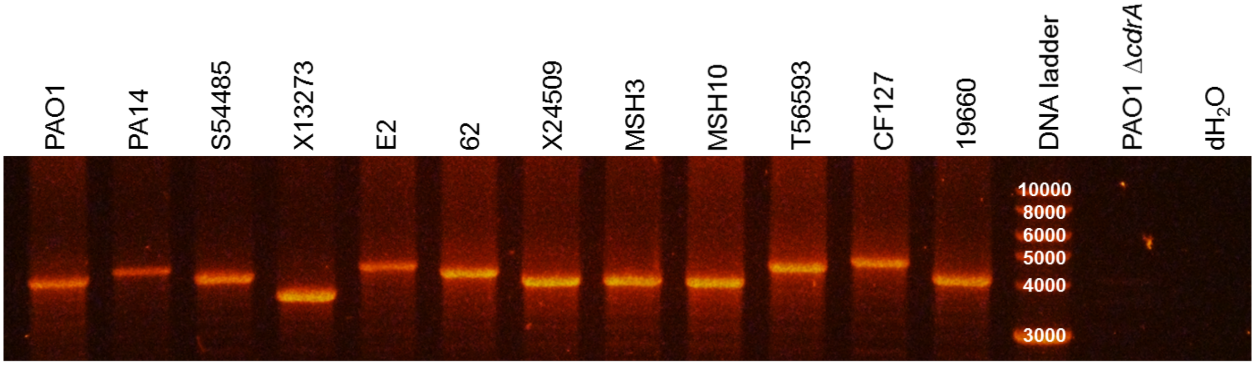

Supplemental Fig 2. PCR amplification of the *cdrA*-repeat region. The PCR products were run on a 0.8% TBE agarose gel. The expected PAO1 *cdrA* amplicon size is 4048 bp.

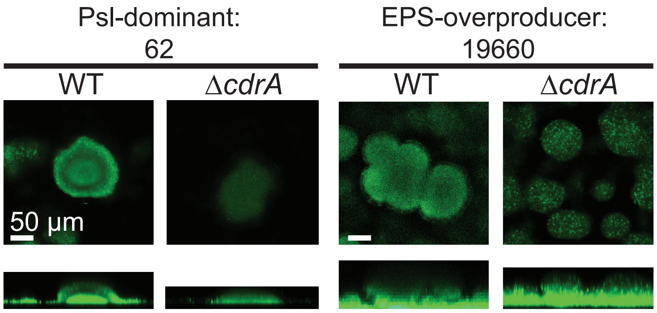

Supplemental Fig 3. Biofilm aggregation in flow-cells. Representative flow-cell biofilms of isolates "62" and "19660" stained with Syto9 and imaged using CLSM.

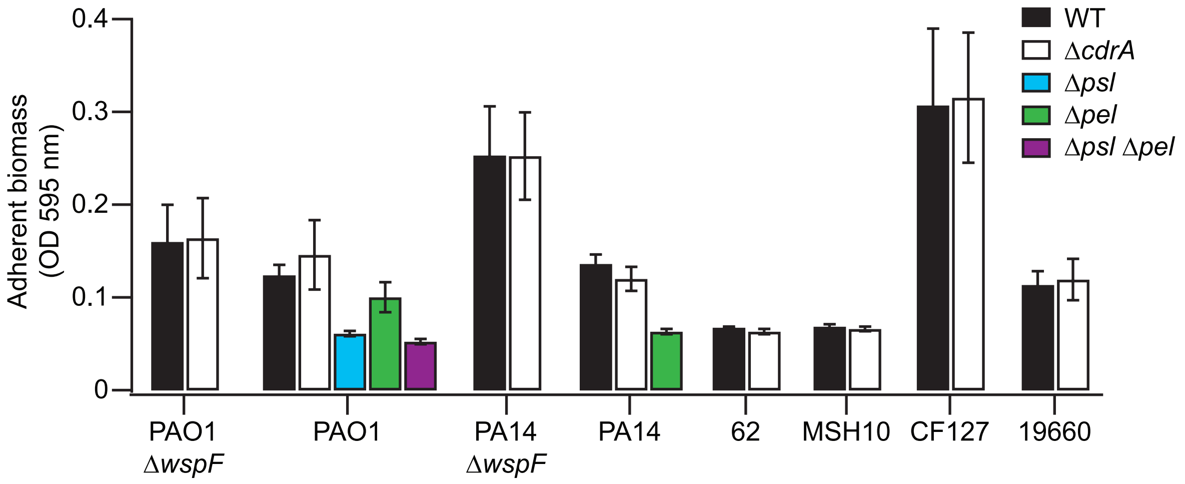

Supplemental Fig 4. CdrA does not significantly impact static biofilm formation under these conditions. Static biofilm formation of wild-type (WT) and mutant strains was measured by crystal violet staining. Data represent the mean of four biological replicates (6 wells per replicate) and error bars indicate standard deviation.

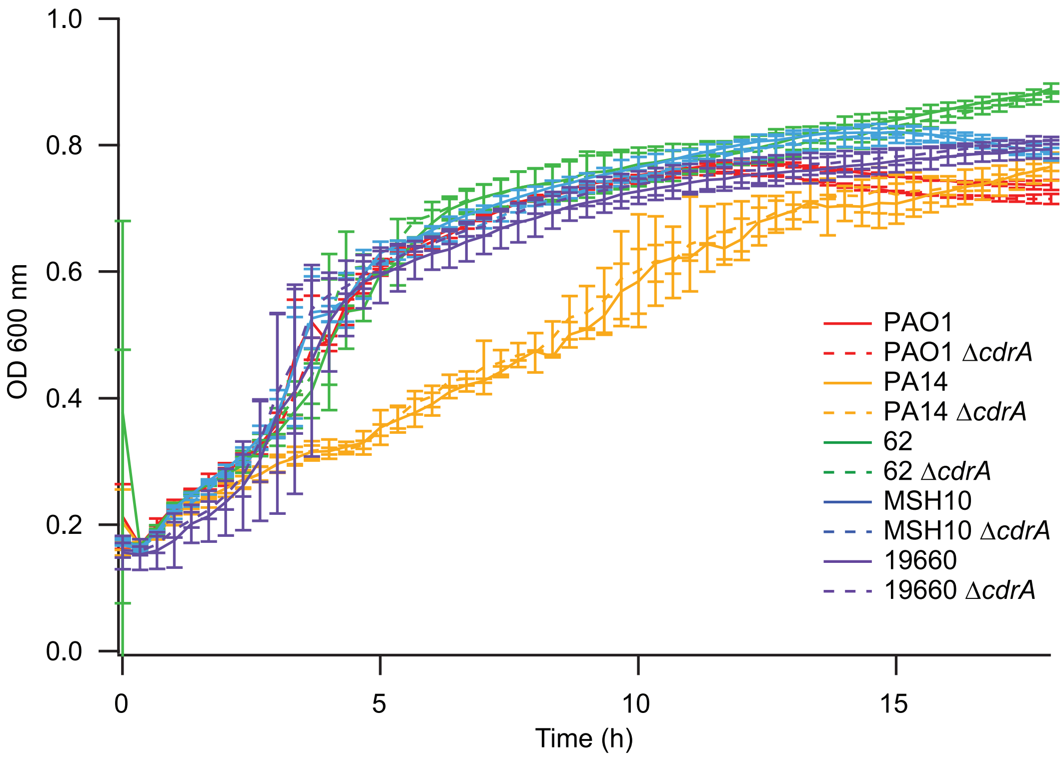

Supplemental Fig 5. Growth curve of isolates and their isogenic Δ*cdrA* mutants. Isolates were cultured in 96-well plates with at 37°C with TSB as the growth medium. Data represent the mean of two biological replicates with three wells per replicate, and error bars indicate standard deviation.

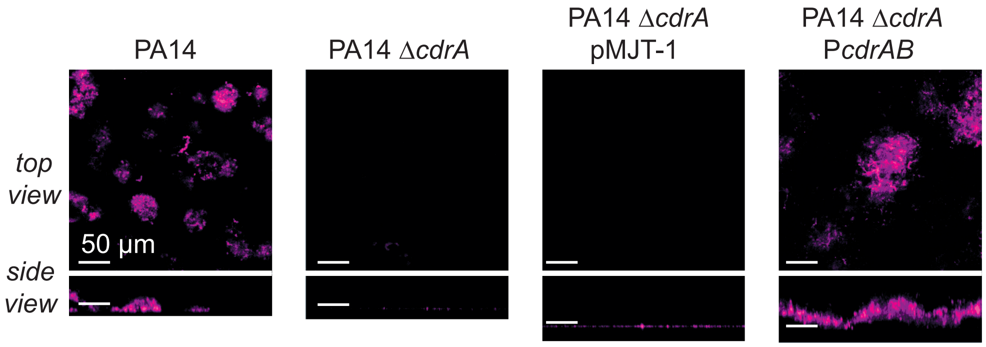

Supplemental Fig 6. The biofilm aggregate defect of PA14 Δ*cdrA* can be complemented. In contrast to PA14, PA14 Δ*cdrA* did not form biofilms in flow-cells. To determine if the Δ*cdrA* biofilm defect could be complemented, PA14 Δ*cdrA* was transformed with the inducible plasmid pBAD*cdrAB* (P*cdrAB*) or the empty vector control (pMJT-1). Flow-cell biofilms were cultured in 1% TSB. For PA14 Δ*cdrA* pMJT-1 and PA14 Δ*cdrA* P*cdrAB*, 0.05% (w/v) L-arabinose was included in the culture medium. After 3 days of growth, the biofilms were stained with Syto62 and imaged using CLSM. Aggregates were observed for PA14 Δ*cdrA* P*cdrAB*. No aggregates were observed for the negative control PA14 Δ*cdrA* pMJT-1.

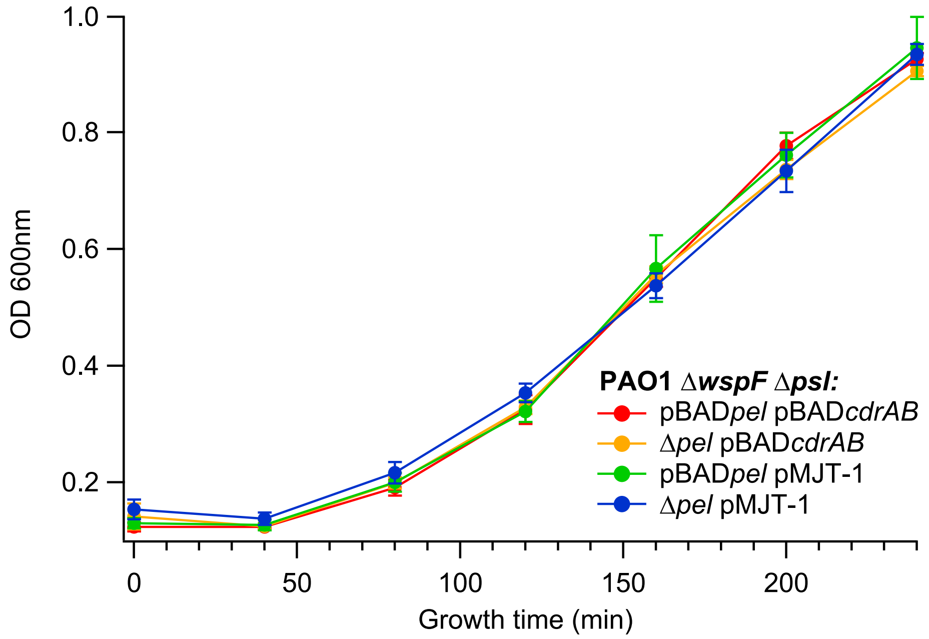

Supplemental Fig 7. Growth curve of strains used in the liquid-culture aggregation assay. We performed a growth curve of uninduced cultures grown under the same conditions (37°C with Jensen's as the growth medium) and encompassing the time used in the aggregation assay. We did not observe a difference in between the strains. It was necessary to use the uninduced cultures because the aggregates in the induced cultures could not be readily broken up for accurate OD readings. The average values of three separate cultures are displayed on the plot, and the error bars were calculated through the standard deviation of each of the replicate samples.

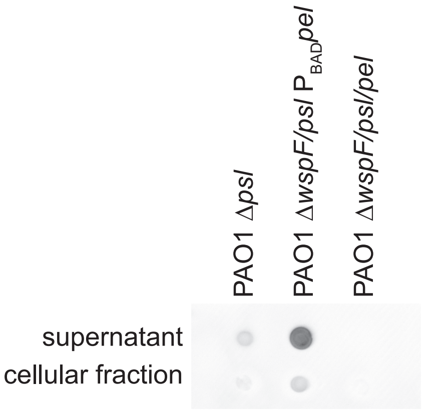

Supplemental Fig 8. Immunoblot of Pel shows that PAO1 Δ*wspF/psl* P_{BAD}*pel* produces more Pel than PAO1. The levels of the EPS Pel were probed using anti-Pel antisera.

REFERENCES

Borlee BR, Goldman AD, Murakami K, Samudrala R, Wozniak DJ, Parsek MR. 2010. *Pseudomonas aeruginosa* uses a cyclic-di-GMP-regulated adhesin to reinforce the biofilm extracellular matrix. Molecular Microbiology 75:827–842.

Jennings LK, Storek KM, Ledvina HE, Coulon C, Marmont LS, Sadovskaya I, Secor PR, Tseng BS, Scian M, Filloux A, Wozniak DJ, Howell PL, Parsek MR. 2015. Pel is a cationic exopolysaccharide that cross-links extracellular DNA in the *Pseudomonas aeruginosa* biofilm matrix. Proceedings of the National Academy of Sciences 112:11353–11358.

Reichhardt C, Wong C, Passos da Silva D, Wozniak DJ, Parsek MR. 2018. CdrA Interactions within the *Pseudomonas aeruginosa* biofilm matrix safeguard it from proteolysis and promote cellular packing. mBio 9:e01376–18.
